# 770 Increasing SOAR Participation in a Burn Center through a Social Work Team Approach

**DOI:** 10.1093/jbcr/irac012.323

**Published:** 2022-03-23

**Authors:** Margaret M Dines, Margaret Spotts, Laura Madsen, Arek J Wiktor

**Affiliations:** University Of Colorado Hospital, Aurora, Colorado; UC Health Burn and Frostbite Center - Anschutz Medical Campus, Denver, Colorado; University of Colorado Hospital, Aurora, Colorado; University of Colorado Burn Center, Aurora, Colorado

## Abstract

**Introduction:**

Peer support has long been used in Burn Centers through organized support groups and programs like Phoenix Society for Burn Survivor’s SOAR (Survivors Offering Assistance in Recovery) individual peer support, often led by Social Workers (SW). The addition of an ambulatory SW in partnership with an inpatient SW allowed our Burn Center to continue participation in the group and individual peer support offerings despite the simultaneous COVID-19 pandemic. We sought to examine participation in support services using our dual SW model.

**Methods:**

Prior to 2020, our ABA verified Burn Center only had an inpatient SW who was able to engage our admitted patient population to support services. With the addition of an ambulatory SW, we have been able to target support group participation in the larger ambulatory population. Our dual SW model allows for continued recruitment to our support services; meeting the patient’s needs at each stage of recovery. This tag team approach, coupled with increased use of technology driven by the pandemic, has shown to increase the average number of participants. We reviewed our support group participation and attendance over the past 2 years.

**Results:**

In calendar year 2020 with one SW facilitator, only 14 virtual support groups were held with an attendance average of 4-5 participants and 22 individual peer support visits. In only the first 9 months of calendar year 2021 with the addition of a second SOAR trained SW, 18 support groups were completed virtually with new inpatient, in-person participation. The participation rate of now bi-monthly support groups has increased to 9 participants per support group average. At this continued rate, we expect to serve 216 attendees per year through support group. In this same 9-month span 51 SOAR individual peer support visits (12 in-person and 39 virtual), were conducted. Which is an increase from the 22 total peer support visits facilitated in 2020.

**Conclusions:**

An additional SOAR trained SW to our Burn Center has increased participation and availability of support groups and individual peer support visits. Peer support promotes socialization and can provide healing for the burn patients and their families in a meaningful and profound way. Burn centers must continue to prioritize the role of the clinical SW to ensure programs such as SOAR support group and individual peer support can be facilitated to ensure an environment that fosters psychological and emotional healing.

